# Comparison of different technologies for the decipherment of the whole genome sequence of *Campylobacter jejuni* BfR-CA-14430

**DOI:** 10.1186/s13099-019-0340-7

**Published:** 2019-12-16

**Authors:** Lennard Epping, Julia C. Golz, Marie-Theres Knüver, Charlotte Huber, Andrea Thürmer, Lothar H. Wieler, Kerstin Stingl, Torsten Semmler

**Affiliations:** 1 NG1-Microbial Genomics, Robert Koch Institute, Nordufer 20, 13353 Berlin, Germany; 2 National Reference Laboratory for Campylobacter, Department of Biological Safety, Federal Institute for Risk Assessment, Berlin, Germany; 3 Advanced Light and Electron Microscopy, Robert Koch Institute, Berlin, Germany; 4 Methodology and Research Infrastructure, Robert Koch Institute, Berlin, Germany

**Keywords:** *Campylobacter jejuni*, Long read sequencing, Hybrid assemblies, Assembler comparison, Antibiotic resistance

## Abstract

**Background:**

*Campylobacter jejuni* is a zoonotic pathogen that infects the human gut through the food chain mainly by consumption of undercooked chicken meat, raw chicken cross-contaminated ready-to-eat food or by raw milk. In the last decades, *C. jejuni* has increasingly become the most common bacterial cause for food-born infections in high income countries, costing public health systems billions of euros each year. Currently, different whole genome sequencing techniques such as short-read bridge amplification and long-read single molecule real-time sequencing techniques are applied for in-depth analysis of bacterial species, in particular, Illumina MiSeq, PacBio and MinION.

**Results:**

In this study, we analyzed a recently isolated *C. jejuni* strain from chicken meat by short- and long-read data from Illumina, PacBio and MinION sequencing technologies. For comparability, this strain is used in the German PAC-CAMPY research consortium in several studies, including phenotypic analysis of biofilm formation, natural transformation and in vivo colonization models. The complete assembled genome sequence most likely consists of a chromosome of 1,645,980 bp covering 1665 coding sequences as well as a plasmid sequence with 41,772 bp that encodes for 46 genes. Multilocus sequence typing revealed that the strain belongs to the clonal complex CC-21 (ST-44) which is known to be involved in *C. jejuni* human infections, including outbreaks. Furthermore, we discovered resistance determinants and a point mutation in the DNA gyrase (*gyrA*) that render the bacterium resistant against ampicillin, tetracycline and (fluoro-)quinolones.

**Conclusion:**

The comparison of Illumina MiSeq, PacBio and MinION sequencing and analyses with different assembly tools enabled us to reconstruct a complete chromosome as well as a circular plasmid sequence of the *C. jejuni* strain BfR-CA-14430. Illumina short-read sequencing in combination with either PacBio or MinION can substantially improve the quality of the complete chromosome and epichromosomal elements on the level of mismatches and insertions/deletions, depending on the assembly program used.

## Background

*Campylobacter jejuni* is a Gram-negative bacterium that colonizes a wide range of hosts as part of the natural gut microbiota [1]. It is frequently found in farm animals such as chicken and cattle or in wild birds. While consuming undercooked poultry meat, unpasteurized milk or cross-contaminated ready-to-eat food it can colonize the human gut and cause an infectious gastroenteritis together with diarrhea, fever and cramps [2].

Over the past two decades the incidence of *Campylobacter* infections has continued to increase worldwide and has become a dangerous threat to public health. To date, campylobacteriosis is the most common bacterial cause of food-born infections in high income countries, with costs amounting to 2.4 billion euros each year for the public health system and lost productivity in the European Union [3].

The BfR-CA-14430 strain was first isolated during the zoonosis monitoring program, in which distinct matrix–pathogen combinations were collected by federal state laboratories. The strain was isolated from a German chicken meat sample in August 2016 using ISO 10272-1:2006 [4]. Since this strain was chosen to serve as a fresh field strain for the German research consortium PAC-CAMPY, we analyzed characteristics of BfR-CA-14430, like antibiotic resistance and virulence factors. In addition, we gained a deeper insight into whole genome sequencing and the impact of various assembly programs, including different hybrid assemblers on various combinations of long and short read sequencing technologies. This revealed a complete chromosomal sequence as well as one closed plasmid sequence.

## Methods

### Bacterial isolation and initial characterization

BfR-CA-14430 was isolated in the framework of the zoonosis monitoring program 2016 from chicken meat according to ISO 10272-1:2006. Species identification was performed by Real-time PCR according to Mayr et al. [5]. The multi locus sequence type was determined by Sanger sequencing (PubMLST) and confirmed by whole-genome sequencing (WGS). The flagellin subunit A (*flaA)* type was Sanger sequenced [6], typing was done according to PubMLST (pubmlst.org) and compared with the outcome of the WGS analysis. BfR-CA-14430 was cultured either on Columbia blood agar (Oxoid) or in brain heart infusion (Oxoid) at 42 °C under microaerobic conditions (5% O_2_, 10% CO_2_) and cells were harvested by centrifugation.

### Antimicrobial resistance determination by microdilution

BfR-CA-14430 was pre-cultured on Columbia blood agar for 24 h at 42 °C under microaerobic atmosphere. Broth microdilution susceptibility testing was performed according to VET06 and M45-A [7]. 2–8 × 10^5^ CfU/ml were inoculated into cation-supplemented Mueller Hinton broth (TREK Diagnostic Systems, UK) supplemented with 5% fetal calf serum (PAN-Biotech, Germany), into the European standardized microtiter EUCAMP2 or EUVSEC plate formats (TREK Diagnostic Systems). Samples were incubated for 48 h at 37 °C under microaerobic conditions. Minimal inhibitory concentrations (MIC; [mg/l]) were semi-automatically analyzed using the Sensititre Vizion system and the SWIN-Software (TREK Diagnostic Systems). Epidemiological cut-off values for resistance determination were based on the European Committee on Antimicrobial Susceptibility Testing (EUCAST.org), if already defined for *C. jejuni* or, alternatively, for *Salmonella* (EUVSEC plate format).

### Genomic DNA extraction and sequencing

DNA extraction for Sanger MLST analyses was performed with GeneJET Genomic DNA Purification Kit (Thermo Fisher Scientific). DNA for WGS was prepared using the MagAttract HMW Genomic Extraction Kit (Qiagen) (for PacBio and Illumina sequencing) and QIAamp DNA Mini Kit (Qiagen) for MinION sequencing and further concentrated by precipitation with 0.3 M sodium acetate pH 5 and 0.7 volume isopropanol at room temperature for 30 min. After centrifugation and washing of the precipitate with 70% ice-cold ethanol, the DNA was dissolved in Tris buffer pH 7.5. The quality of the DNA was evaluated by spectral analysis (NanoDrop Spectrophotometer, Thermo Fisher Scientific, USA) and the concentration was fluorimetrically quantified to be 110 ng/µl by Qubit 3.0 Fluorometer (dsDNA BR Assay Kit; Invitrogen, USA). DNA was additionally controlled for lack of sheering products < 20 kb on a 0.8% agarose gel. Sequencing was performed on a MiSeq sequencer (MiSeq Reagent Kit v.3; Illumina Inc., San Diego, CA, USA), using the Library Preparation kit Nextera XT (Illumina Inc., San Diego, CA, USA) resulting in 300-bp paired-end reads and an average coverage of around 100-fold. Furthermore, size selection was performed using 10 K Blue Pippin and DNA was sequenced with Single Molecule Real-Time (SMRT) Sequencing Technology on a PacBio RS II by GATC Biotech AG (Konstanz, Germany) as well as with long read sequencing on Oxford Nanopore MinION (Oxford, UK) (Library-Kit: Rapid Barcoding Kit (SQK-RBK004), Flowcell: 1D R9.4, without size selection, base calling with albacore v2.1.0) in order to compare these three techniques for establishing a complete genome with epichromosomal elements. Total amounts of extracted DNA of 1 ng, 5 µg and 400 ng was used as starting material for sequencing by MiSeq, PacBio or MinION, respectively. A general overview of the raw data from the different sequencing machines can be found in Table 1.

**Table 1:** Summary of the raw output from Illumina, MinION, and PacBio sequencing technologies

Technology	Number of reads	Total number of bases	Median read length	Calculated mean genome coverage
Illumina MiSeq	658,314	165,840,055	285	98×
PacBio RS II	88,482	802,118,168	9065	475×
MinION	61,960	737,318,830	8073	436×

### Genome assembly and annotation

Sequencing reads obtained from the MiSeq sequencer were (i) assembled by the SPAdes v3.12 [8] and plasmidSPAdes [9] assembler or (ii) used to correct long read data. Furthermore we used the CLC Genomics Workbench v12.0.1 as well as an assembly from the PacBio in-house pipeline HGAP v3.0 [10] and Flye v2.5 [11] for the PacBio long read assemblies. The assembly based on MinION raw reads was only performed by Flye v2.5. All assemblers were run with default settings. To generate an optimal assembly and derive a closed genome sequence we tested various de novo hybrid assembly tools on different combinations of short and long reads (Unicycler v0.4.7 [12] and wtdbg2 v2.1 [13]). Unicycler first creates a draft genome assembly with SPAdes v3.12 and connects the contigs only afterwards by using the long reads from PacBio or MinION. Wtdbg2, on the other hand, first assembles the long reads and corrects the assembly afterwards by mapping the short reads against the genome. Long reads were mapped to the genomes by minimap2 v2.14 [14]. The different combinations of short and long reads used for each tool are shown in Table 2. In order to annotate the genomes, a custom-made database of 137 complete genomes of *C. jejuni* downloaded from NCBI (Additional file 1: Table S1) was built and used as a Genus-specific BLAST database for Prokka v1.13 [15].

**Table 2:** Summary of the assembler performance based on different sequencing technologies

Index	Data	Assembler	#Contigs	#bp total length	#Chromosomal contigs; #bp	#plasmid; #bp	Insertions, deletions and SNPs	Covered by illumina reads	Sequence identity of *flaA*
A	Illumina	SPAdes	30	1,666,0451	30; 77,674 (N50)^a^	Cannot be directly detected	0^b^	99.9^c^	100
B	PacBio	HGAP	2	1,733,585	1; 1,668,827	1; 64,758	155	99.46	100
C	PacBio	Flye	2	1,687,377	1; 1,645,611	1; 41,766	255	99.99	100
D	PacBio	CLC	2	1,688,161	1; 1,646,367	1; 41,794	253	99.97	100
E	PacBio + Illumina	Unicycler	3	1,684,748	2; 1,631,764/ 11,212	1; 41,772	0	99.9	100
F	PacBio + Illumina	wtdbg2	3	1,693,078	2; 1,644,895/ 6,442	1; 41,741	47	99.65	100
G	MinION	Flye	2	1,720,675	1; 1,678,003	1; 42,673	24,439	99.36	99.6
H	MinION + Illumina	Unicycler	2	1,687,752	1; 1,645,980	1; 41,722	20	99.94	100
I	MinION + Illumina	wtdbg2	5	1,672,121	4; 1,648,160/ 15,620/12,211/6,130	1/41,957	169	98.15	100

^a^Quality of draft genomes can be measured by the N50 value

^b^Illumina paired-end data is taken as ground truth for identification of SNPs, insertions and deletions

^c^As result of the scaffolding process, performed by the SPAdes assembler, contigs with known distance, but unknown sequence content, are connected by “N”s. Thus, the SPAdes assembly is not covered by Illumina data by 100%

### Assembly comparison and in silico analysis

The assembled genomes were compared by the progressive Mauve algorithm [16] to detect major structural differences. Single nucleotide polymorphisms (SNPs) were detected by mapping the Illumina paired-end reads against the assemblies by bowtie2 v4.8.2 [17] with the end-to-end sensitive mode. SNPs, insertions and deletions were counted within an allele frequency of at least 75% at positions with a minimum of 10 reads by freebayes v.1.2.0 [18] according to Illumina short reads. The multi locus sequence typing (MLST) was performed by a BLAST based pipeline (https://github.com/tseemann/mlst) to identify the allele variants of the seven housekeeping genes (*aspA*, *glnA*, *gltA*, *glyA*, *pgm*, *tkt* and *uncA*). Point mutations conferring antibiotic resistance or individual antibiotic resistance genes were revealed by ResFinder 3.0 [19] (CGE, DTU, Lyngby, DK; https://cge.cbs.dtu.dk/services/ResFinder/).

### Quality assurance

In order to perform an in-silico control for contamination within the sequenced DNA, Illumina short reads were adapter trimmed with Flexbar [20] and all reads were taxonomically classified as *C. jejuni* by Kraken v2.0.6 [21]. Taxonomic classification of the long reads could identify 3.71% of Human related DNA within the PacBio read, which has been removed. Assembly completeness and contamination was controlled with checkM v. 1.0.18 [22].

## Results

### Antimicrobial resistance profile of BfR-CA-14430

The minimal inhibitory concentration (MIC) of different antibiotics was determined using the broth microdilution susceptibility approach (CLSI). Using the standard EUCAMP2 plate format, which is used for screening of *C. jejuni* resistance during zoonosis monitoring, the strain showed resistance against ciprofloxacin, nalidixic acid and tetracycline but was sensitive towards erythromycin, gentamicin and streptomycin. We extended the antimicrobial substances and applied the EUVSEC plate format, usually tested with *Salmonella* and *Escherichia coli* isolates. As *C. jejuni* is intrinsically resistant against most of the cephalosporine antibiotics, it was expected that strain BfR-CA-14430 was also resistant against cefotaxime, cefoxitime, cefepime, ceftazidime. The cephalosporine cefoperazone is used as a selective supplement in ISO 10272:2017 in mCCDA (modified charcoal-cefoperazone agar) and Bolton broth. Besides, the strain revealed natural resistance against trimethroprim due to the absence of the target dihydrofolate reductase (FolA). However, MIC values for sulfamethoxazole were 16 mg/l, rendering the strain sensitive, on the basis of a cut-off value used for *Salmonella* of 64 mg/l. Furthermore, resistance against ampicillin was also seen with MIC values > 64 mg/l, while MIC values for meropeneme, ertapeneme and colistin were 0.25 and 0.5 and 2 mg/l, respectively. BfR-CA-14430 was fully susceptible to chloramphenicol, tigecycline, azithromycin and imipeneme, with MIC values below the lowest test concentration.

### Genomic features of the strain BfR-CA-14430

Using multilocus sequence typing, the strain BfR-CA-14430 was identified as sequence type ST-44 which belongs to the clonal complex CC-21 that is frequently found in human infections and well known to cause *C. jejuni* outbreaks [23]. The complete genome sequence, assembled from MinION and Illumina reads by Unicycler, consists of one chromosome of 1,645,980 bp covering 1,665 coding sequences (CDSs), including *bla*_OXA-61_ (Cj0299 in NCTC 11168) that encodes for a beta-lactam resistance gene [24] and a point mutation in the gyrase subunit A (gyrA) (T86I) [25], conferring resistance against (fluoro-)quinolones. All AMR genes or AMR associated SNPs could be detected within the hybrid assembly as well as in the Illumina paired-end reads. Additionally, the genome has 44 transfer RNA (tRNA) genes, 9 ribosomal RNA (rRNA) genes forming three identical operons consisting of 16S, 23S and 5S subunits and an overall GC content of 30.4%. The chromosome harbors the virulence factors *cdtA*, *cdtB*, *cdtC*, coding for the cytolethal distending toxin, the gene encoding the fibronectin-binding protein CadF and the *Campylobacter* invasion antigens CiaB and CiaC. Genes encoding the monofunctional α 2,3-sialyltransferase CstIII and the *N*-acetylneuraminic acid biosynthesis proteins NeuA1, NeuB1 and NeuC1 are present for lipooligosacharide (LOS) sialylation, which was shown to be linked to Guillain–Barré syndrome onset [26, 27]. The conserved capsule biosynthesis *kpsC* and *kpsF* genes flank the variable capsule locus of approximately 26 kb, belonging to the Penner type HS1 complex [28]. Besides, the *pseA-I* genes involved in flagellar protein glycosylation [29] were detected on the chromosome. Furthermore, the strain carries a single circular plasmid of 41,772 bp including 46 CDSs. Among these genes the plasmid carries a *tetO* gene for tetracycline resistance as well as *virB2-11* and *virD4* genes encoding for a putative type IV secretion system (T4SS), for conjugative DNA transfer between *Campylobacter* strains [30]. The plasmid showed 93% identity and 98% coverage with plasmid pTet from *C. jejuni* strain 81–176 (45,025 bp) (CP000549) and 98% identity and 97% coverage with plasmid pMTVDSCj16-1 (42,686 bp) from *C. jejuni* strain MTVDSCj16 (NZ_CP017033.1) that carry type IV secretion systems and *tetO* genes as well [31]. By mapping of Illumina paired-end reads, plasmid pMTVDSCj16-1 was covered by 97% with 99% identity and 611 SNPs. Two regions of 600 bp and 1113 bp were not covered by the Illumina reads. However, read mapping was not able to detect a region 927 bp containing a CDS that can also be found in pTet-M129 (NZ_CP007750.1) [32] of *C. jejuni* strain M129 (NZ_CP007749.1) and pRM5611 (NZ_CP007180.1) from C. coli strain RM5611 (NZ_CP007179.1).

**Fig. 1:**
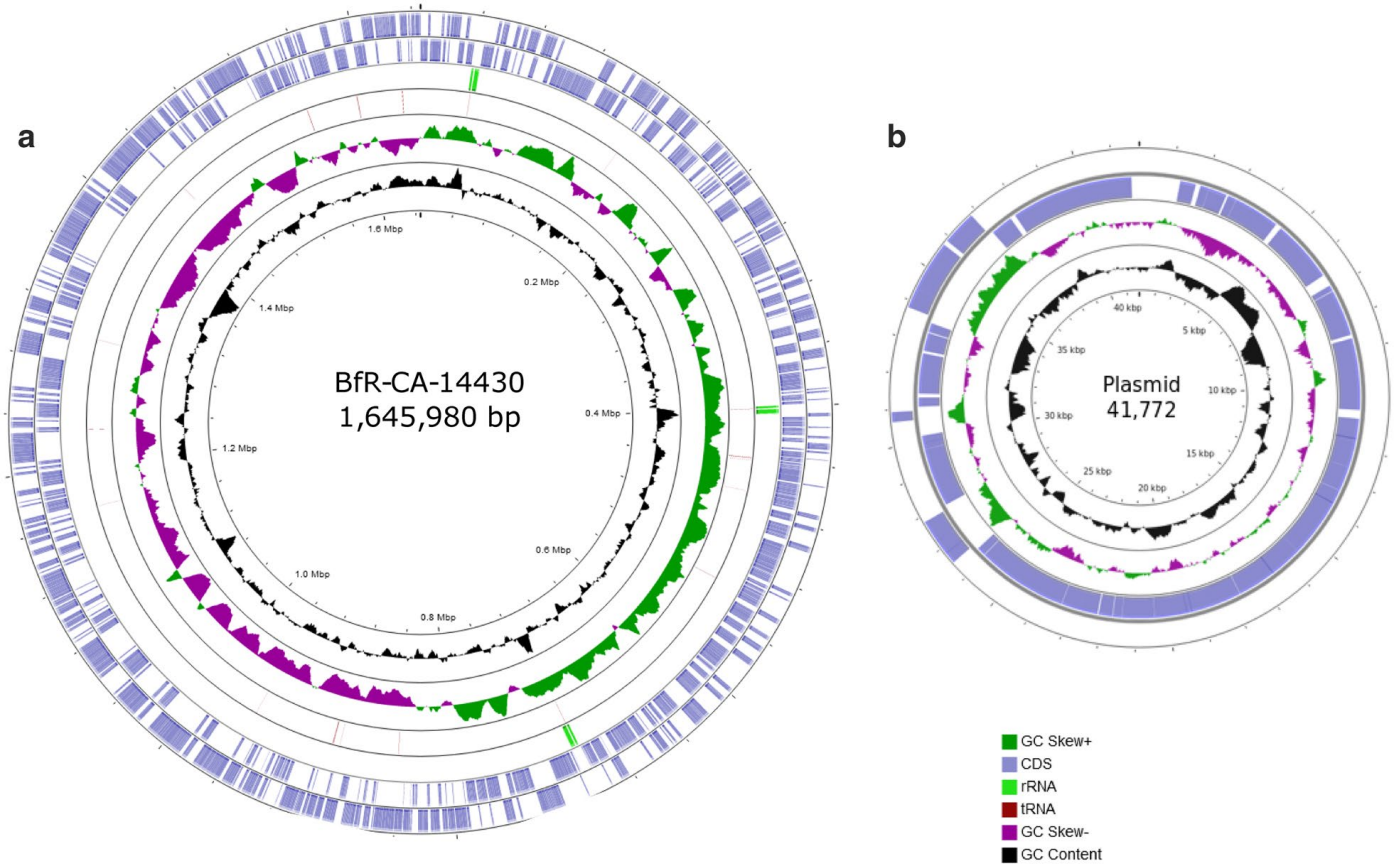
Genome map, generated by CGView [33], of chromosomal DNA a) and plasmid DNA b) from *C. jejuni.* BfR-CA-14430. Circles form outside to inside showing: (1,2) coding regions (light blue) predicted on forward (outer circle) and reverse strands (inner circle); (3) tRNAs (dark red); (4) rRNAs (light green); (5) regions above (green) and below (purple) the average GC skew; (6) GC content (black) and (7) DNA coordinates

The genomic structure and annotation of the chromosome and plasmid are visualized in Fig. 1 and can be accessed at the National Center for Biotechnology Information (NCBI) database with the accession numbers CP043763 and CP043764.

### Assembly comparison

**Fig. 2:**
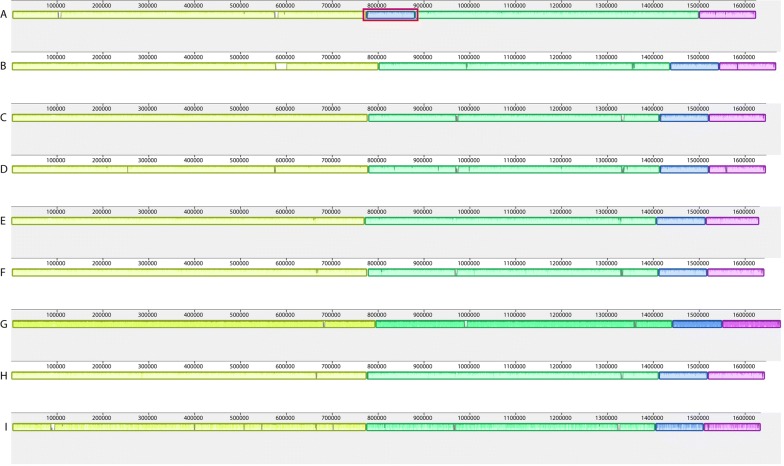
Progressive Mauve Alignment of chromosomal genomes generated by different assemblers. The Misassembly made by SPAdes is marked by the red square. Assemblies are index by alphabetic letters as shown in Table 2. Color coded blocks indicating homology between the genomes

Whole genome comparison of all assemblies showed that each assembler created one chromosome of around 1.6 Mb as well as one plasmid of around 42 kb while using PacBio or MinION long reads in combinations with Illumina short reads (Table 2). Gel electrophoresis of extracted DNA from BfR-CA-14430 suggested the occurrence of chromosomal and plasmid DNA. All long read assembler reconstructed the chromosomal genome in one single contig without large structural variations (Fig. 2). Reads from MinION and Illumina that were assembled by Unicycler resulted in a circular genome. However, some tools generated small extra contigs (Table 2): The combination of Illumina and PacBio data as well as MinION with Illumina data as input to the wtdgb2 assembler generated contigs that were later identified by BLAST to be part of the chromosomal sequence of the strain. With the advantage of using long reads, one misassembly inside a repeat region in the SPAdes assembly based on the Illumina short reads was discovered (Fig. 2). Additionally, we were able to identify the Sanger sequenced *flaA* gene with a sequence identity of 100% in most of the cases (Table 2). The MinION assembly generated with Flye did not reach 100% sequence identity, due to the high number of SNPs within this assembly.

**Fig. 3:**
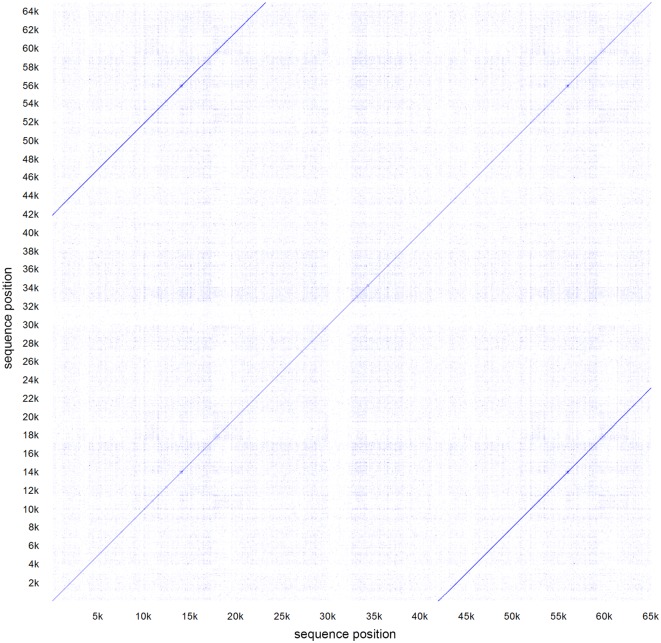
The dotplot shows a global alignment of the plasmid sequence, generated from PacBio reads by HGAP (Table 2B), against itself. This revealed one dark blue diagonal line in the middle from start to end of the sequence as well as two additional dark blue lines showing up in the top left and bottom right part of the plot. Those lines show a repeat from 42 to 65 kb and 1 to 23 kb, respectively. Therefore, the sequence is identical in the first 23 kb as well as the last 23 kb and indicates it as a large repeat region that is likely to be cause through an assembly error

Furthermore, all tools assembled a plasmid with a size of around 42 kb, except from the PacBio in-house pipeline that created a 64 kb plasmid. By performing a global alignment against itself and generating a dotplot we could show a large repeat region between the first and the last 20 kb in the circular sequence that obviously originates from an assembly error (Fig. 3). Plasmid assemblies produced by Unicycler were found to be circularized, while using PacBio as well as MinION data. Identification of plasmid sequences by plasmidSPAdes, revealed 9 from 3 components. Besides the ca. 42 kb plasmid described earlier, the 8 other sequences could be identified as part of the chromosomal DNA by BLAST from strain BfR-CA-11430 as well as in several closed genomes from Additional file 1: Table S1. Those assembled DNA fragments mainly have their origin in low coverage or repeat regions, which cannot be resolved by short reads and is known to lead to misassemblies in plasmidSPAdes [9].

Standalone assemblies of long read data from MinION generated the overall correct structure of the genome and the plasmid, but a lot of small insertions, deletions and SNPs were additionally created (Table 2). The assembly of MinION raw reads contains more than 25,000 SNPs, which is around 100 times more compared to assemblies of PacBio reads with HGAP and Flye. However, by combining MinION with Illumina data the SNP count decreased to only 20 SNPs. The assembly from HGAP or Flye based on PacBio raw reads contains 155 SNPs and 255 SNPs respectively whereas the combination of PacBio and Illumina contains 0 SNPs.

The final chromosomal assembly of MinION and Illumina reads is covered by 95×, 424× and 375×, whereas the plasmid sequence is covered by 204×, 291× and 3021× from Illumina, PacBio and MinION reads. Genome completeness was calculated to be at 99.36% and contamination was predicted to be 0.15%.

## Conclusion

Here, we describe the *C. jejuni* strain BfR-CA-14430 that carries a beta lactamase and tetracycline resistance gene as well as potential virulence factors that might play a role in human gut infection. Furthermore we compared multiple hybrid assembly methods based on different sequencing technologies. This revealed that the combination of long reads with short reads decreases the SNP rate in de novo assemblies to a large extent. In general, using a combination of long and short reads as input to the Unicycler assembler resulted in accurate and closed chromosomal and plasmidal sequences for our data. However, assemblies based only on PacBio reads, seem to be highly accurate and can also be used without being polished by Illumina data.

## Supplementary information

**Additional file 1: Table S1.** *Campylobacter jejuni* Genomes used to build the Prokka Database

## Data Availability

The completed genome sequence of *BfR-CA-14430* has been deposited into GenBank database with accession number CP043763 (chromosome) and CP043764 (plasmid), respectively. Raw read data from Illumina Miseq, PacBio RS II and Oxford Nanopore MinION is available at NCBI with SRA accession number PRJNA562653.
